# Correlation analysis of the puncture-side bone cement/vertebral body volume ratio and bone cement leakage in the paravertebral vein in vertebroplasty

**DOI:** 10.1186/s12891-022-05135-w

**Published:** 2022-02-26

**Authors:** Tao Gao, Zhi-Yu Chen, Tao Li, Xu Lin, Hai-Gang Hu, De-Chao Yuan, Jun Zeng, Chao Wu

**Affiliations:** 1 Orthopaedics of Zigong Fourth People’s Hospital, Zigong, Sichuan China; 2 Respiratory Medicine of Zigong Fourth People’s Hospital, Zigong, 643000 Sichuan China

**Keywords:** Vertebroplasty, Cement leakage in the paravertebral vein, Volume of puncture-side bone cement, Volume of vertebral body, Puncture-side bone cement/vertebral volume ratio

## Abstract

**Objectives:**

To explore the influencing factors of bone cement leakage in the paravertebral vein after vertebroplasty for the treatment of osteoporotic vertebral compression fractures (OVCFs) and to determine the correlation between the puncture-side bone cement/vertebral body volume ratio and bone cement leakage in the paravertebral vein.

**Methods:**

This was a retrospective analysis of 495 patients (585 vertebral bodies) with OVCFs treated from August 2018 to May 2021 in our hospital. The patients’ postoperative CT data were imported into Mimics software, and the three-dimensional(3D) reconstruction function was used to calculate the bone cement volume (BCV), puncture-side bone cement volume (PSBCV), and vertebral body volume (VBV); the bone cement/vertebral body volume ratio (BCV/VCV%) and puncture-side bone cement/vertebral body volume ratio (PSBCV/VCV%) were additionally calculated. Sex, Age, Body mass index(BMI), Bone density, BCV, PSBCV, VBV, BCV/VCV%, and PSBCV/VCV were compared between the leakage group and the non-leakage group. Logistic regression analysis was used to assess the correlations between the factors that statistically significantly differed between the two groups and the presence of leakage in the paravertebral veins. A receiver operating characteristic (ROC) curve was used to determine the diagnostic value of the PSBCV/VCV% and to obtain the optional cut-off value.

**Results:**

A total of 102 males and 393 females with an average age of 72.89 (52 ~ 93) years were included in our study. There were 57 cases of cement leakage (59 vertebral bodies) in the paravertebral vein. There were 438 patients (526 vertebral bodies) without paravertebral cement leakage. Univariate analysis showed that the differences in sex, bone density, PSBCV, and PSBCV/VCV% between the two groups were statistically significant (*P* < 0.05). Logistic regression analysis showed that there were correlations between sex, bone density, and PSBCV/VCV% and the presence of paravertebral cement leakage (*P* < 0.05). The ROC curve showed that the area under the curve of the PSBCV/VCV% for the diagnosis of cement leakage in the paravertebral vein was greater than 0.65, and *P* < 0.05, indicating a diagnostic value. The best cut-off point for the diagnosis of paravertebral cement leakage with the PSBCV/VCV% was 13.68%, with a sensitivity of 84.7% and specificity of 37.8%.

**Conclusion:**

Sex, bone density, and PSBCV/VCV% are risk factors for cement leakage in the paravertebral veins after vertebroplasty for the treatment of OVCFs; the PSBCV/VCV% is strongly associated with paravertebral venous leakage, and the optimal PSBCV/VCV% is 13.68%. When the PSBCV/VCV% exceeds the optimal value, the risk of cement leakage in the paravertebral vein becomes significantly increased.

## Introduction

Vertebroplasty has been used to treat osteoporotic vertebral compression fractures for decades, but the problem of bone cement leakage still cannot be prevented [1–3]. The types of bone cement leakage that occur after vertebroplasty include intraspinal canal leakage, intervertebral disc leakage, paravertebral soft tissue leakage, and paravertebral vein leakage, depending on the location of leakage [4]. Bone cement leakage in the paravertebral vein often does not cause clinical symptoms [5–7], but if it is not detected promptly, bone cement can return to the pulmonary blood vessels through the vena cava and cause pulmonary embolism, which can be life-threatening and cause severe complications [8–10]. Therefore, preventing paravertebral cement leakage is particularly important. A review of the literature shows that the current research on bone cement leakage in the paravertebral vein mainly focuses on the puncture site, vertebral body blood vessel distribution, and BCV/VCV% [11–15]. During vertebroplasty, bone cement is dispersed unevenly in the vertebral body. The BCV is usually dispersed more evenly on the puncture side than on the contralateral side, and cement leakage in the paravertebral vein usually occurs in the puncture-side vertebral body, but there have been no relevant reports about the correlations between PSBCV, PSBCV/VCV% and cement leakage in the paravertebral vein. This study included new indicators, PSBCV and PSBCV/VCV%, based on the previously considered influencing factors to further explore the influencing factors of cement leakage in the paravertebral vein after vertebroplasty and to determine the correlation between PSBCV/VCV% and bone cement leakage in the paravertebral vein.

## Materials and methods

### General information

The study was approved by the institutional review board. From August 2018 to May 2021, 495 patients (585 vertebral bodies) were treated with vertebroplasty for osteoporotic vertebral compression fractures in our hospital. All patients had a complete preoperative bone density, CT, and magnetic resonance imaging(MRI) data and postoperative CT data. The preoperative and postoperative imaging examinations of all patients were completed under the same scanner in our hospital. The CT scanner model isSIEMENS SOMATOM Force.

### Inclusion and exclusion criteria

The inclusion criteria for the patients were as follows: 1) severe low back pain that was aggravated with postural changes, such as turning, was not relieved after systematic conservative treatment, and was associated with tenderness upon local spinous process tapping; 2) MRI scans showing a compressive fracture of the vertebral body at the painful site and vertebral bone marrow oedema; and 3) the absence of symptoms of spinal cord and nerve injury.

The exclusion criteria were as follows: 1) age younger than 50 years old; 2) infection at the fracture site or uncontrolled systemic infection; 3) abnormal blood coagulation function that was uncurable; 4) a severe fracture causing puncture difficulty; 5) postoperative pathological examination results showing no fractures; 6) severe cardiopulmonary dysfunction and other absolute surgical contraindications.

### Surgical methods

All the surgeries were performed by qualified and experienced attending physicians. The bone cement materials and puncture systems were provided by the same manufacturers (Via Andrea Doria). The vital signs of the patients were routinely monitored. The patients were placed in a prone position, the skin was sterilized with towels, and C-arm fluoroscopy was used to determine the position of the pedicle on the puncture side. Lidocaine 1% was used for layer-by-layer anaesthesia. A 0.5 cm skin incision was made under the guidance of C-arm fluoroscopy, and the vertebral body was entered through the unilateral pedicle puncture. The lateral fluoroscopic puncture tip was located on the posterior wall of the vertebral body. When the puncture reaches the anterior 1/3 of the vertebral body, The puncture working channel was created, and a small amount of vertebral bone tissue was removed for medical examination. When the bone cement had the consistency of toothpaste, the bone cement was slowly injected into the vertebral body under C-arm fluoroscopy, and lateral fluoroscopy was stopped when the bone cement approached the posterior wall of the vertebral body. After the bone cement was completely cured, the working sleeve was rotated and pulled out, and the sterile auxiliary materials were bandaged after the incision was disinfected. X-ray and CT scans were taken on the second day after surgery.

### Imaging analysis

**Fig. 1:**
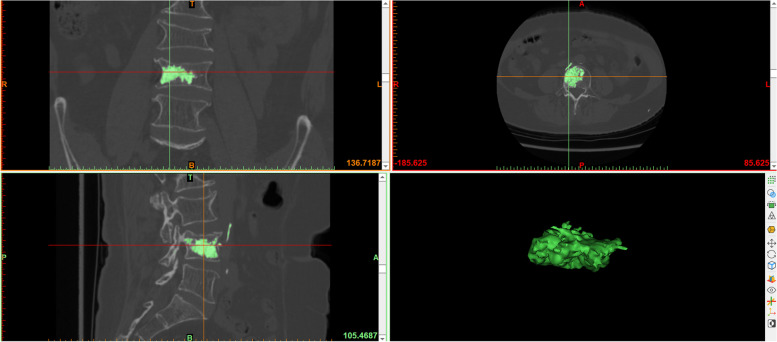
Calculate the volume of bone cement by Mimics software

**Fig. 2:**
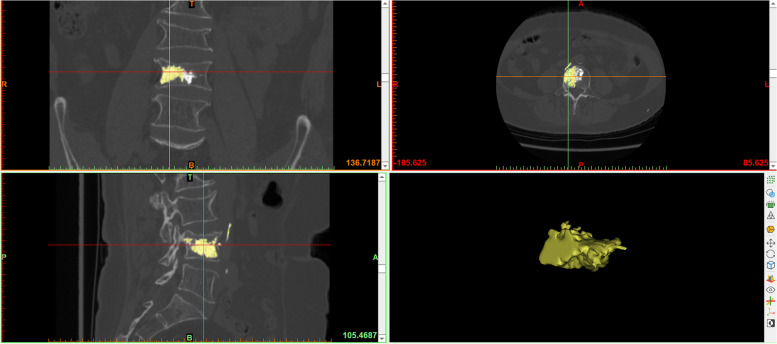
Calculate the volume of puncture side bone cement by Mimics software

**Fig. 3:**
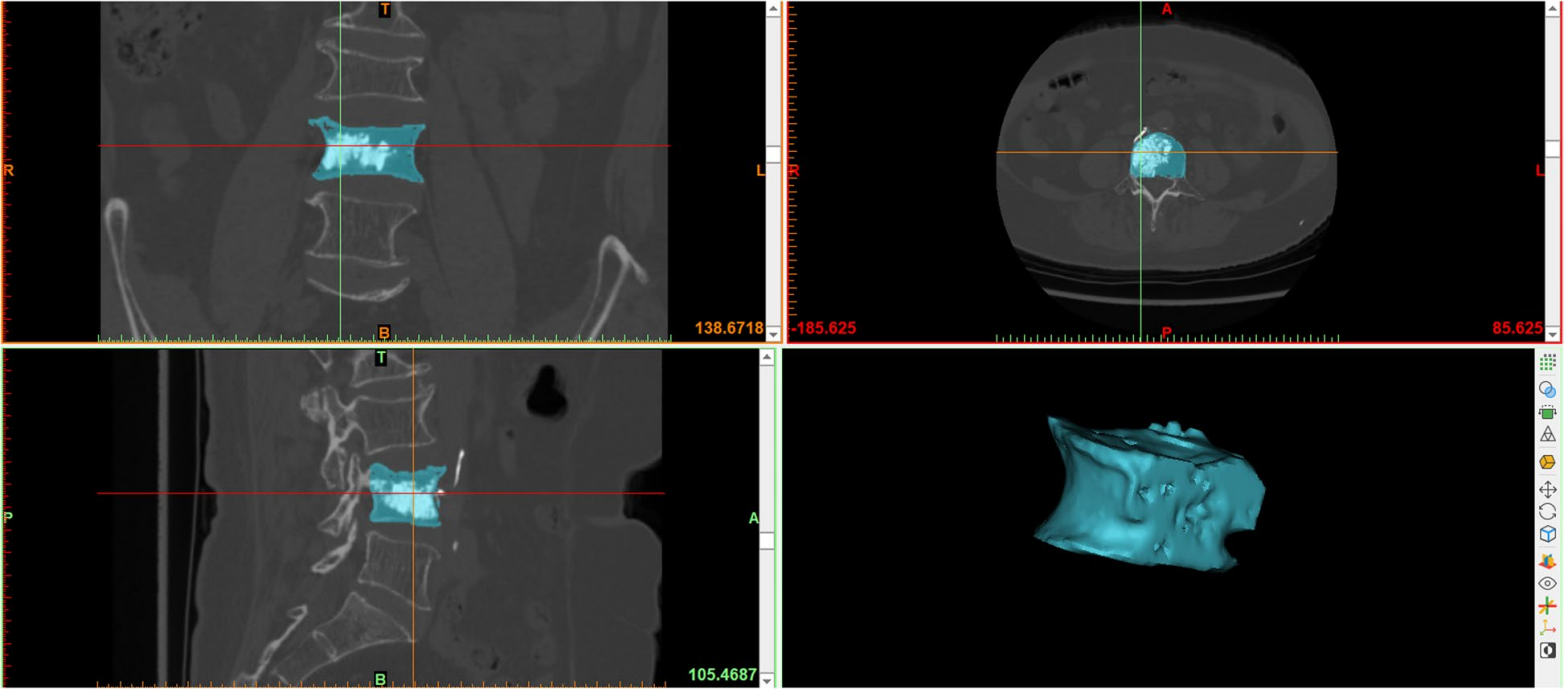
Calculate the volume of vertebral body by Mimics software

Mimics software was used to calculate the volume of the bone cement and vertebral body: the patients’ CT data were imported into Mimics 21.0 software, and the segmentation threshold was set by the threshold segmentation function. Since the bone cement was much more dense than bone, the bone cement threshold could be segmented quickly (1500 ~ 3071 Hounsfield unit); the segmentation result was saved as a mask, and the bone cement in the vertebral body of the target segment was selected and saved as a mask using the crop mask function. The 3D image of the bone cement was reconstructed by the 3D mask reconstruction function, and the volume value from the 3D image was recorded, which was accurate to 0.01 cm^3^ (Fig. 1). The midpoint of the upper and lower vertebral endplates in the coronal position was marked, the vertebral body was divided into left and right parts, the volume of the cement included in the half of the vertebral body of the access point (puncture side) was defined puncture side bone cement. The puncture side bone cement mask was selected with the mask cutting function, and the PSBCV was determined using the same method as described above (Fig. 2). The threshold segmentation function was used to segment the bone threshold (226 to 3071 Hounsfield units) and save it as a mask. The connections between the target vertebral body and the vertebral pedicle, ribs, and leaking bone cement were separated at each cross-section with the edit mask function (the separation point between the vertebral body and pedicle was the vertical leading edge of the pedicle), the gap-filling function was used to fill the gap in the vertebral body, and finally, the complete vertebral body mask was separated with the 3D mask reconstruction function to reconstruct a 3D image of the vertebral body and record the volume value from the 3D image (Fig. 3).

A calculator was used to calculate the BCV/VCV% and PSBCV/VCV%. Two resident physicians jointly observed the postoperative lateral X-ray and three-dimensional reconstruction of the CT scan and selected the paravertebral veins leaking into the vertebral body.

### Statistical methods

Statistical analysis was carried out using SPSS 18.0 statistical software. The normally distributed measurement data are represented by ‾*x* ± *s*. The comparisons of the indicators between the two groups were performed by the t-test and χ2 test. Binary logistic multiple regression analysis was used to assess the correlations. *P* < 0.05 was considered statistically significant. A ROC curve was generated, and the area under the curve and cut-off value of PSBCV/VCV% were calculated to assess the correlation between the PSBCV/VCV% and the presence of bone cement leakage in the paravertebral vein.

## Results

A total of 102 males and 393 females with an average age of 72.89 (52 ~ 93) years were included in our study. Among these patients, there were 57 cases of cement leakage in the paravertebral vein (59 vertebral bodies), and 2 cases showed leakage from 2 vertebral paravertebral veins. There were 438 patients (526 vertebral bodies) with no paravertebral cement leakage, 370 patients with-single segment fractures, 55 patients with double-segment fractures, 8 patients with three-segment fractures, 3 patients with 4-segment fractures, and 2 patients with a 5-segment fracture. The leakage rates of the paravertebral veins in different parts of the vertebral body are shown in Table 1. The CT images of all patients were of good quality, and the three-dimensional images were reconstructed completely. The volume of bone cement, the volume of bone cement on the puncture side, and the volume of the vertebral body are shown in Table 2.

**Table 1:** Vertebral segment distribution and paravertebral vein leakage rate in two groups

Vertebral body	Leakage group (n)	Non-leakage group (n)	Leakage rates (%)
T5	0	2	0
T6	1	3	33.33
T7	2	11	18.18
T8	3	22	13.64
T9	5	20	25.00
T10	5	43	11.63
T11	9	66	13.63
T12	8	107	7.48
L1	11	103	10.68
L2	8	68	11.76
L3	6	34	17.65
L4	1	30	3.33
L5	0	17	0
Total	59	526	11.22

**Table 2:** Univariate analysis of factors of bone cement leakage in the paravertebral vein

Factor	Leakage group	Non-leakage group	*t*/*χ*^*2*^	*P*
Sex(M/F)	18/41	97/429	4.891	0.027
Age(Y)	71.59 ± 7.49	73.05 ± 8.19	-1.302	0.447
BMI	22.97 ± 3.13	23.08 ± 3.56	-0.228	0.081
Bone density	-2.72 ± 0.94	-3.23 ± 1.15	3.261	0.020
BCV(cm^3^)	5.31 ± 0.92	5.30 ± 0.85	0.128	0.575
PSBCV(cm^3^)	4.06 ± 0.90	3.59 ± 0.74	4.439	0.048
VBV(cm^3^)	24.57 ± 1.81	24.59 ± 1.86	-0.098	0.654
BCV/VBV%	22.52 ± 2.84	21.45 ± 2.48	0.210	0.125
PSBCV/VBV%	16.44 ± 3.17	14.53 ± 2.43	5.523	0.016

### Univariate analysis of the factors affecting bone cement leakage in the paravertebral vein

The univariate analysis results showed that there were statistically significant differences in sex, bone density, PSBCV, and PSBCV/VCV% between the two groups (*P* < 0.05). There was no significant difference in age, BMI, BCV, VBV, or BCV/VBV% between the two groups (*P* > 0.05) (Table 2).

### Logistic regression analysis of the factors affecting the leakage of bone cement in paravertebral veins

Logistic regression analysis showed that sex, bone density, and PSBCV/VCV% were correlated with the presence of paravertebral cement leakage, but PSBCV was not significantly correlated with the presence of paravertebral cement leakage (Table 3).

**Table 3:** Binary logistic regression analysis of the factors affecting bone cement leakage in the paravertebral vein

Factor	B	S.E	Wald	*P*	Exp(B)	95% C.I.for EXP(B)	
						Lower	Upper
Sex	-0.683	0.324	4.444	0.035	0.505	0.268	0.953
PSBCV	-0.810	0.472	2.944	0.086	0.445	0.176	1.122
PSBCV/VBV%	0.474	0.141	11.240	0.001	1.607	1.218	2.120
Bone density	0.291	0.132	4.849	0.028	1.338	1.033	1.734

### Comparison of the ROC curve and area under the curve of various factors for the diagnosis of paravertebral cement leakage

**Fig. 4:**
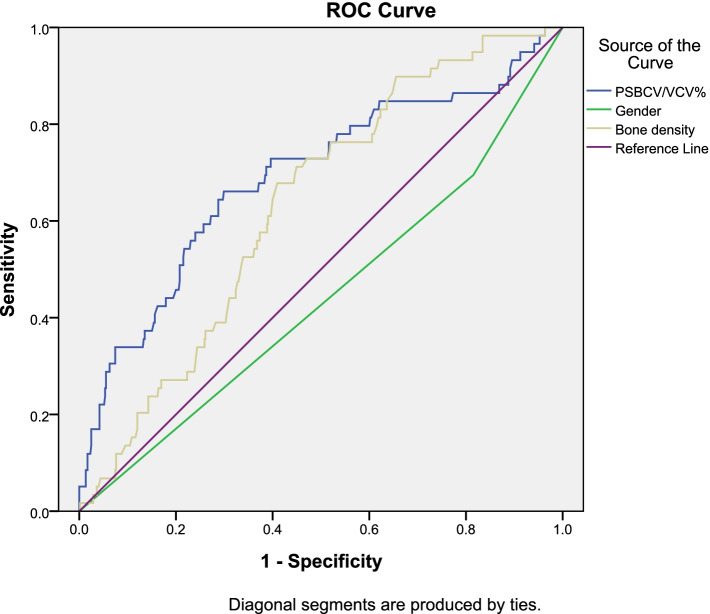
The ROC curve for diagnosis of paravertebral cement leakage by factors

The ROC curve of the diagnosis of paravertebral cement leakage was generated in the logistic regression analysis of sex, bone density, and PSBCV/VCV% (Fig. 4). The area under the PSBCV/VCV% curve for the diagnosis of paravertebral cement leakage was > 0.65, and *P* < 0.05, indicating diagnostic value (Table 4).

**Table 4:** Area under the ROC curve for the diagnosis of paravertebral cement leakage

Factor	AUC (Area)	*P*	*95%CI*	
			Lower	Upper
PSBCV/VBV%	0.690	< 0.001	0.610	0.769
Sex	0.440	0.129	0.359	0.521
Bone density	0.633	0.001	0.567	0.699

### The best cut-off points of the various factors for the diagnosis of paravertebral cement leakage

The best cut-off point for the PSBCV/VCV% for the diagnosis of paravertebral cement leakage was 13.68%, with a sensitivity of 84.7% and specificity of 37.8% (Table 5).

**Table 5:** The best cut-off point of the puncture-side bone cement/vertebral volume ratio for the diagnosis of paravertebral cement leakage

Factor	Sensitivity	Specificity
PSBCV/VBV%(13.68%)	0.847	0.378

## Discussion

The incidence of venous cement leakage caused by vertebroplasty varies by its location in the body, and paravertebral cement leakage is the most common type. The vertebral body's venous system is composed of internal and external vertebral veins, vertebral body veins, and connecting veins. The internal vertebral veins are radial, the anterior and lateral sides of the vertebral body are connected to the anterior vertebral vein through a small venous channel, and the rear is connected to the communication branch behind the ligamentum flavum [16]. The vertebral venous system lacks a venous valve, so when bone cement enters the vertebral venous system, it easily moves to a distant place and eventually enters the pulmonary blood vessels [17]. After bone cement enters the blood vessels, it activates the coagulation system and promotes the production of pulmonary capillary thrombin; then, a large number of platelets accumulate, and the blood becomes hypercoagulable. In severe cases, disseminated intravascular coagulation(DIC) may occur. These factors are likely to cause pulmonary embolism [18]. Most pulmonary embolisms caused by bone cement leakage do not cause clinical symptoms [5–7, 19, 20], which is why most patients do not undergo chest radiographs after vertebroplasty. Studies have shown that bone cement emboli in the lungs are mostly concentrated in the blood vessels around the lungs, rather than in specific lobar tissues, and the position and shape of the bone cement emboli can remain unchanged for a long time after surgery [21]. Although most bone cement emboli do not cause clinical symptoms, a small number can cause severe pulmonary embolism [8–10, 18]. Hsieh MK et al. [22] studied 9 patients with pulmonary embolism caused by paravertebral vein leakage after vertebroplasty. Therefore, determining how to avoid bone cement vein leakage is particularly important.

The univariate analysis results in this study showed that there was a correlation between bone density and paravertebral vein leakage. Xie W et al. [23] also showed that vertebral bone density is an independent risk factor for bone cement leakage, which may be related to different bone densities having different vertebral pressures. Heini PF et al. [24] found that the higher the bone density was, the higher the pressure in the vertebral body during vertebroplasty. Aebli N et al. [25] performed vertebroplasty on the vertebral body of sheep and monitored the leakage of bone cement under different pressure states. The authors found that increased pressure in the vertebral body can cause bone cement leakage in extradural and paravertebral veins. Because the venous wall in the vertebral body is thin, when the intramedullary pressure exceeds the bearing range when bone cement is injected, bone cement particles are squeezed into the vein and cause paravertebral vein leakage. Yan J et al. [26] reported it can reduce the cement leakage rate by decreasing the intravertebral pressure. Patients with higher bone density have greater resistance during vertebroplasty, and higher pressure is often required to inject the bone cement. In addition, the higher the bone density is, the greater the number of bone trabeculae per unit area. The smaller the gap between them is, the smaller the volume of bone cement that can be accommodated between the trabecular bones. Therefore, the same volume of bone cement needs more space for diffusion, and the risk of bone cement leakage into the paravertebral veins increases accordingly. In a study by Liu J et al. [27], the same volume of bone cement, 3 ml, was injected into the same segment. The results showed that the higher the bone density was, the smaller the diffusion space of the vertebral bone cement. Therefore, it can be considered that there is a certain relationship between bone density and paravertebral vein leakage. When the bone density is high, it is necessary to closely observe whether there is also paravertebral cement leakage during vertebroplasty.

The results of this study show that there is a correlation between sex and paravertebral vein leakage, and male patients are more prone to exhibiting paravertebral vein leakage. Zhu SY et al. [12] also showed that male patients are more prone to exhibiting bone cement leakage than are female patients. The reason for this finding may be that male patients tend to have a higher bone density, which requires a higher pressure to inject the bone cement.

The univariate and binary logistic regression analysis in this study showed that PSBCV/VBV% is a risk factor for paravertebral cement leakage, and the PSBCV/VBV% area under the ROC curve was greater than 0.65, indicating diagnostic value. Previous studies mainly focused on the effects of BCV and BCV/VBV% on bone cement leakage. Multiple studies have shown that the greater the amount of bone cement that is injected, the higher the risk of venous bone cement leakage [11, 12, 22, 28]. Zhu SY et al. [12] found that to avoid bone cement leakage, a BCV of less than 3.5 ml in the thoracic spine and a BCV of less than 4 ml in the lumbar spine are safe. Fu Z et al. [29] also showed that the amount of BCV was closely related to the leakage of cement in paravertebral veins. Unfortunately, this study did not give a recommended value for the amount of BCV. To date, no research on the PSBCV has been conducted. This study showed that the BCV is not a risk factor for paravertebral cement leakage, but the PSBCV is closely related to leakage in the paravertebral vein. Bone cement is often unevenly distributed in the vertebral body during vertebroplasty. Usually, the bone cement is more distributed on the puncture side than on the contralateral side. Therefore, leakage of the bone cement in the paravertebral vein usually appears in the puncture side vein, so this study shows that the PSBCV has a larger effect on paravertebral cement leakage than does the BCV. Because the VBV of each patient is greatly affected by age, sex, race, the fracture segment, etc., it is not appropriate to simply consider the effect of the BCV on bone cement leakage, and the BCV/VBV% is not affected by the above factors. Therefore, in recent years, the effect of BCV/VBV% on bone cement leakage has gradually become a widely studied topic [13, 15, 30–32]. Jin YJ et al. [32] found that the risk of pulmonary embolism significantly increased when the BCV/VCV% exceeded 21% at the T11-L1 level and recommended that the largest BCV/VCV% that should be used to relieve pain and prevent leakage complications is 11.65%. Kwon HM et al. [31] showed that the best BCV/VCV% during vertebroplasty was 27.8%. However, in both studies, the BCV was calculated on the basis of the amount of bone cement injected intraoperatively, and the VBV was calculated using a mathematical model. However, due to the leakage of bone cement and residual bone cement through the puncture sleeve, the actual BCV in the vertebral body is not equal to the volume of injected bone cement, and the VBV calculated by the mathematical model is not equal to the actual VBV, so the BCV/VBV% cannot be calculated accurately in this way. In this study, the postoperative CT data of the patients were imported into Mimics software, which can accurately calculate the actual BCV and the real VBV and then accurately calculate the BCV/VBV%. Sun H et al. [13] also accurately calculated the BCV and VCV with software and pointed out that the optimal BCV/VCV% during vertebroplasty was 19.78%, with a sensitivity of 86.60% and specificity of 51.50%, and the risk of bone cement leakage increased as the BCV/VCV% increased. In this study, it was found that the BCV/VCV% had no obvious correlation with paravertebral cement leakage, while the PSBCV/VCV% was a risk factor for paravertebral cement leakage. The best cut-off point for the PSBCV/VCV% for the diagnosis of paravertebral cement leakage was 13.68%, with a sensitivity of 84.7% and specificity of 37.8%. Because paravertebral vein leakage usually occurs in the punctured-side lateral vein, this study shows that the PSBCV/VCV% can be used to detect paravertebral cement leakage.

## Conclusion

Sex, bone density, and PSBCV/VCV% are risk factors for cement leakage in the paravertebral vein; PSBCV/VCV% has the largest influence on paravertebral cement leakage, and the optimal PSBCV/VCV% is 13.68%. When the PSBCV/VCV% exceeds the optimal value, the risk of cement leakage in the paravertebral vein becomes significantly increased.

## Data Availability

All data generated or analysed during this study are included in this published article.

## Abbreviations

OVCFs: Osteoporotic vertebral compression fractures
CT: Computed tomography
MRI: Magnetic resonance imaging
3D: Three-dimensional
BCV: Bone cement volume
PSBCV: Puncture side bone cement volume
VBV: Vertebral body volume
BCV/VCV%: Bone cement/vertebral body volume ratio
PSBCV/VCV%: Puncture side bone cement/vertebral body volume ratio
BMI: Body mass index
ROC: Receiver operating characteristic
CI: Confidence interval
DIC: Disseminated intravascular coagulation
